# Implementation of Cardiac Stereotactic Radiotherapy: From Literature to the Linac

**DOI:** 10.7759/cureus.13606

**Published:** 2021-02-28

**Authors:** Rachel M Glicksman, Abhishek Bhaskaran, Kumaraswamy Nanthakumar, Patricia Lindsay, Catherine Coolens, Leigh Conroy, Daniel Letourneau, Benjamin H Lok, Meredith Giuliani, Andrew Hope

**Affiliations:** 1 Radiation Medicine Program/Radiation Oncology, Princess Margaret Cancer Centre/University of Toronto, Toronto, CAN; 2 The Hull Family Cardiac Fibrillation Management Laboratory, Peter Munk Cardiac Centre, Toronto General Hospital, University Health Network, Toronto, CAN

**Keywords:** arrhythmia, cardiac, sbrt, sabr, implementation science, knowledge translation

## Abstract

Stereotactic radiotherapy (SBRT) has been applied to treat cardiac arrhythmias, but our institution had not yet implemented this technique. Here, we explain how we used implementation science and knowledge translation to provide cardiac SBRT to a critically ill patient with malignancy-associated refractory ventricular tachycardia. We reviewed the critical factors that enabled the implementation of this urgent treatment, such as the context of the implementation, the characteristics of the intervention, and the stakeholders. These principles can be used by other radiation programs to implement novel treatments in urgent settings, where the gold standard process of planning and developing policies and protocols is not possible.

## Introduction

Recently, multiple case reports and one phase I/II trial described the use of cardiac stereotactic radiotherapy (SBRT) for non-malignant cardiac disease [1-5]. Despite these well-publicized descriptions, our institution had not formally implemented cardiac SBRT and had no first-hand experience with it, although we were in the preliminary stages of developing a cardiac SBRT protocol. Our center was able to urgently implement cardiac SBRT to treat a critically ill patient with malignancy-associated ventricular tachycardia non-responsive to medical and electrical cardioablation [6].

Here, our rapid implementation process is explored from the lens of implementation science to provide guidance for other centers to implement new technologies such as cardiac SBRT on an urgent basis when the gold standard process of planning and developing policies and procedures (P&Ps) or treating patients enrolled in clinical trials are not possible.

## Technical report

Implementation science

The diffusion of medical research and innovation to reach medical practice is prolonged at approximately 17 years [7]. Therefore, publications of clinical research in journals is not sufficient to translate new knowledge to clinical care [8]. Knowledge translation, the method of closing the gaps from knowledge to practice, is needed to synthesize, disseminate, and exchange knowledge [8]. Furthermore, the success of any healthcare implementation effort is dependent on three components outlined in the normalization process model: the context in which the implementation effort is attempted, the characteristics of the intervention, and the stakeholders [9].

Implementation context

Our practice setting is a single-payer, publicly funded tertiary hospital. The center has expertise in cardiovascular care with a cardiac intensive care unit (CICU), and oncology with a high-volume cancer center with a radiation department having significant SBRT experience. The radiation department has multiple P&Ps to outline competencies and standards in all radiation-related activities in an effort to improve quality of care [10,11]. It can be challenging in this setting to perform urgent de novo treatments for which no P&P exists. However, we were able to extend many of our existing SBRT policies to guide the first cardiac SBRT case due to the similarities in target volumes, organs at risk, and treatment planning technique (volumetric modulated arc therapy).

Characteristics of intervention

**Figure 1:**
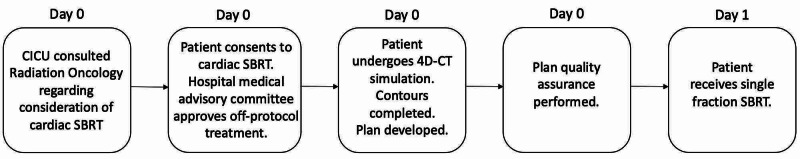
Timeline of implementation from Radiation Oncology consultation to treatment delivery. CICU: cardiac intensive care unit; SBRT: stereotactic radiotherapy; 4D-CT: 4-dimensional computed tomography

The time from consultation to cardiac SBRT delivery was one day (Figure 1). In contrast to most patients who receive cardiac SBRT, this patient did not have a history of ventricular tachycardia or an implantable cardiac defibrillator (ICD). Therefore, risk mitigation strategies (e.g., magnetic resonance imaging [MRI], dose to the device, and pre- and post-treatment ICD interrogation] were not necessary [12].

Simulation

Simulation was performed as per departmental lung SBRT protocol [6]. Because this patient was critically ill, she was on cardiac monitors and monitored by CICU personnel throughout simulation and treatment. Stable patients would likely not require this intensive monitoring.

Target Delineation

**Figure 2:**
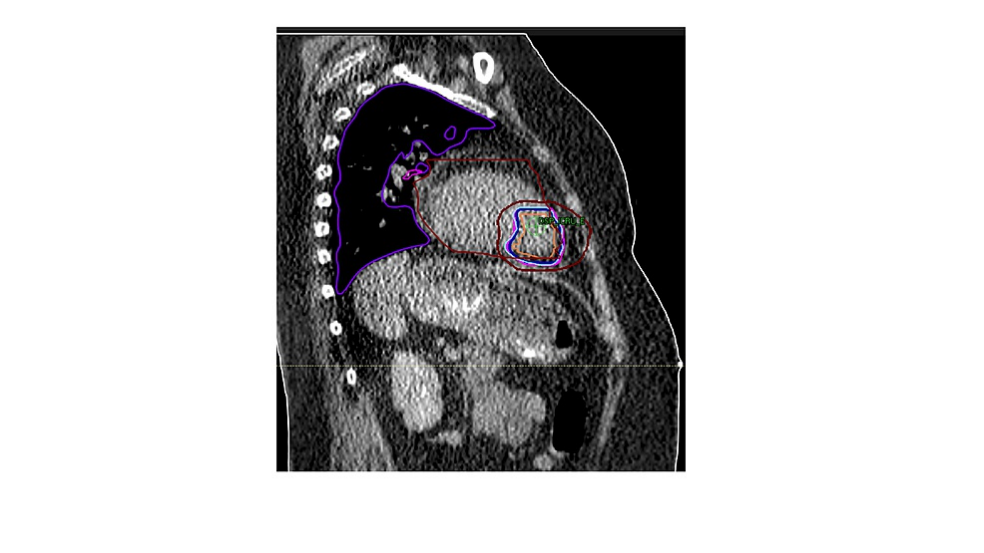
Sagittal representative slice of the cardiac SBRT plan. Contours: Orange: ITV; light blue: PTV; maroon: heart; purple: left lung Isodoses: Dark blue: 100% isodose line (25 Gy); purple: 95% isodose line (23.75 Gy); brown: 50% isodose line (12.5 Gy) SBRT: stereotactic radiotherapy; ITV: internal target volume

Target delineation was performed by a multi-disciplinary team of Radiation Oncologists and Electrophysiologists based on diagnostic and simulation computed tomography (CT) (contoured on maximal inhalation, maximal exhalation, and free breathing sequences), echocardiogram, cine MRI, and an electroanatomic map using the CARTO®3 system (Biosense Webster Inc., CA, USA), using principles from lung SBRT with adjustment of volumes based on patient- and cardiac-specific factors [6]. A representative slice from the planning CT scan with contours and isodose lines is shown in Figure 2.

Motion Management

To assess and account for physiologic target motion in determining the internal target volume (ITV), respiratory-correlated 4D-CT, echocardiogram, and cine MRI, in which multiple images at a single location in the heart were taken throughout the cardiac cycle to assess target and cardiac motion, were used to create an ITV. On the day of the treatment, a dynamic multi-frame volumetric cine was obtained [6] as an additional patient-specific ITV quality assurance (QA) measure. As cardiac SBRT is performed frequently and institutions gain experience with expected variations in cardiac motion, this additional measure will likely become unnecessary. A cone beam CT (non-4D) for soft-tissue matching was performed in the treatment unit prior to treatment delivery. Further details regarding target delineation and motion management are detailed in a clinical case report [6].

Treatment Planning

Similar to lung SBRT, the plan was created on the maximal exhalation image sequence. Although we routinely use flattening filter beams for lung SBRT, in this case a flattening filter-free (FFF) beam was used to reduce overall treatment time [13], minimizing the extent of possible patient movement [14], and allowing for return to the CICU as quickly as possible. FFF beams are a part of our center’s clinical practice, so it was easily adapted to this setting. All routine QA procedures were followed, including peer-review of the contours and treatment plan by a Radiation Oncologist and ArcCHECK by a Medical Physicist. Target coverage and organ at risk dose constraints are detailed in Table 1.

**Table 1:** Target and organ at risk dose-volume metrics. PTV: planning target volume; GTV: gross tumor volume; Gy: Gray

Target or organ at risk	Dose-volume metric achieved in the treatment plan	Dose-volume metric goal
PTV	D95 = 25.18 Gy	D95 = 25.0 Gy
PTV	D99 = 24.29 Gy	D99 = 22.50 Gy
PTV	Dmax = 27.05 Gy	Dmax = 27.5 Gy
Heart	Dmax = 27.05 Gy	Dmax = 27.5 Gy
Heart minus GTV	Average dose = 5.15 Gy	Average dose = 10 Gy
Heart minus PTV	Average dose = 4.27 Gy	Average dose = 10 Gy
Esophagus	Dmax = 5.32 Gy	Dmax = 25 Gy
Lungs	Average dose = 0.95 Gy	Average dose = 5 Gy
Spinal canal	Dmax = 2.53 Gy	Dmax = 8 Gy
Trachea	Dmax = 0.08 Gy	Dmax = 25 Gy
Bronchus	Dmax = 0.3 Gy	Dmax = 25 Gy

Management of Critically Ill Patients

The hospital Medical Advisory Committee approved this urgent off-protocol treatment on compassionate grounds. Preexisting departmental P&Ps for management of critically ill patients were utilized, formulated on the principle of frequent communication between medical teams.

Stakeholder groups

Numerous stakeholders were involved in this implementation, including Cardiologists, Electrophysiologists, Intensivists, Medical Physicists, Radiation Therapists, Dosimetrists, and Radiation Oncologists. Although the cardiac and radiation groups function within large teams in complex environments daily, the specialties rarely share responsibility for the care of the same patient. To effectively achieve coordinated care between the teams, patient, and her caregivers, we used frequent closed-loop communication, facilitative leadership (a leadership style facilitating goal achievement), and a shared mental model (by which the team performance was improved by all team members having a common understanding of the necessary tasks, what needs to be done, and who is responsible to achieve the tasks) [15]. These factors were critical to recognize the indication for cardiac SBRT, delineate target volumes, and ensure clinical stability throughout radiotherapy. Moving forward, the stakeholders will use the experience acquired during this rapid implementation to streamline the design of P&Ps that will support the scaling up of cardiac SBRT for a larger population of patients.

## Discussion

We were able to urgently deliver cardiac SBRT to a critically ill patient with malignancy-associated ventricular tachycardia through principles of implementation science, knowledge translation, and strong multi-disciplinary teamwork.

Limitations

Some of the factors leading to the rapid implementation of cardiac SBRT in this setting may be specific to our institution (e.g., single-payer, publicly funded tertiary hospital with cardiac and radiotherapy expertise) and may not be applicable in all practice settings.

## Conclusions

We provided cardiac SBRT to a patient with malignancy-associated refractory ventricular tachycardia. Despite never before delivering this treatment, we were able to successfully implement it on an urgent basis through implementation science, knowledge translation, and multi-disciplinary teamwork. These principles can be used by other radiation programs to implement novel treatments in urgent settings, where the gold standard process of planning and developing policies and protocols is not possible.
